# Phospho-regulated *Drosophila* adducin is a determinant of synaptic plasticity in a complex with Dlg and PIP_2_ at the larval neuromuscular junction

**DOI:** 10.1242/bio.20148342

**Published:** 2014-11-21

**Authors:** Simon Ji Hau Wang, Amy Tsai, Mannan Wang, SooHyun Yoo, Hae-yoon Kim, Byoungjoo Yoo, Vincent Chui, Marta Kisiel, Bryan Stewart, Wade Parkhouse, Nicholas Harden, Charles Krieger

**Affiliations:** 1 Department of Molecular Biology and Biochemistry, Simon Fraser University, 8888 University Drive, Burnaby, BC V5A 1S6, Canada; 2 Department of Biomedical Physiology and Kinesiology, Simon Fraser University, 8888 University Drive, Burnaby, BC V5A 1S6, Canada; 3 Department of Biology, University of Toronto at Mississauga, 3359 Mississauga Road, Mississauga, ON L5L 1C6, Canada

**Keywords:** Dlg, Drosophila, Hts, PIP2, Adducin, Neuromuscular junction

## Abstract

Adducin is a ubiquitously expressed actin- and spectrin-binding protein involved in cytoskeleton organization, and is regulated through phosphorylation of the myristoylated alanine-rich C-terminal kinase (MARCKS)-homology domain by protein kinase C (PKC). We have previously shown that the *Drosophila* adducin, Hu-li tai shao (Hts), plays a role in larval neuromuscular junction (NMJ) growth. Here, we find that the predominant isoforms of Hts at the NMJ contain the MARCKS-homology domain, which is important for interactions with Discs large (Dlg) and phosphatidylinositol 4,5-bisphosphate (PIP_2_). Through the use of Proximity Ligation Assay (PLA), we show that the adducin-like Hts isoforms are in complexes with Dlg and PIP_2_ at the NMJ. We provide evidence that Hts promotes the phosphorylation and delocalization of Dlg at the NMJ through regulation of the transcript distribution of the PAR-1 and CaMKII kinases in the muscle. We also show that Hts interactions with Dlg and PIP_2_ are impeded through phosphorylation of the MARCKS-homology domain. These results are further evidence that Hts is a signaling-responsive regulator of synaptic plasticity in *Drosophila*.

## INTRODUCTION

The *Drosophila* neuromuscular junction (NMJ) is the site of contact between motor neuron and muscle, and is stably maintained but remodelled during the growth and development of the fly. To permit these differing functions, the NMJ uses an actin- and spectrin-based cytoskeleton both pre- and post-synaptically, where a number of synaptic proteins modify the cytoskeleton dynamically. One such protein involved in the dynamic responses of the synapse to stimuli in vertebrates is the actin- and spectrin-binding protein adducin, a heteromeric protein composed of α, β and γ subunits that is widely expressed in many cell types including neurons and myocytes (Babic and Zinsmaier, 2011; Bennett and Baines, 2001). The adducins are composed of a globular N-terminal head domain, a neck domain and a C-terminal myristoylated alanine-rich C-terminal kinase (MARCKS)-homology domain containing an RTPS-serine residue which is a major phosphorylation site for protein kinase C (PKC), as well as cAMP-dependent protein kinase (PKA) (Matsuoka et al., 1996). Phosphorylation of adducin in the MARCKS-homology domain inhibits adducin-mediated promotion of actin-spectrin interactions, resulting in cytoskeletal reorganization (Bennett and Baines, 2001).

Multiple studies have demonstrated that the mammalian MARCKS protein, or more specifically its MARCKS effector domain, can bind to and sequester the phosphoinositide, phosphatidylinositol 4,5-bisphosphate (PIP_2_), in artificial lipid vesicles (Arbuzova et al., 2000; Denisov et al., 1998; Dietrich et al., 2009; Gambhir et al., 2004; Glaser et al., 1996; Rauch et al., 2002; Wang et al., 2001; Wang et al., 2002; Zhang et al., 2003). This interaction has been linked to the regulation of the actin cytoskeleton during the growth and branching of dendrites in rat brains, as well as the directed migration of bovine aortic endothelial cells in wound healing assays (Kalwa and Michel, 2011; Li et al., 2008). Notably, it has been proposed that aberrant MARCKS regulation of PIP_2_ signaling may be implicated in the formation of amyloid plaques in Alzheimer's disease (Su et al., 2010). A recent study has also provided evidence that reduced hippocampal levels of MARCKS, and thus PIP_2_, in mice contributes to age-related cognitive loss (Trovò et al., 2013).

As mentioned above, MARCKS binds to PIP_2_ as the MARCKS effector domain carries basic residue clusters that can interact with acidic lipids in the inner leaflet of the cell membrane. By analogy to other MARCKS-homology domain-containing proteins, we hypothesize that phosphorylation of adducin at the RTPS-serine may alter the electrostatic interaction between adducin and phosphoinositides, thus reversing the binding between them and causing translocation of adducin from the membrane to the cytosol (McLaughlin and Murray, 2005; Wang et al., 2002). In this way, adducin might act as a molecular switch in its regulation of synaptic plasticity, with its localization at the synapse controlled by phosphorylation (Babic and Zinsmaier, 2011).

In *Drosophila*, orthologs of adducin are encoded by the *hu-li tai shao* (*hts*) locus, and the Hts protein is present at both the pre- and post-synaptic sides of the larval NMJ where it regulates synaptic development (Pielage et al., 2011; Wang et al., 2011). We previously have shown that Hts interacts with the scaffolding protein Discs large (Dlg), and regulates Dlg localization at the postsynaptic membrane by promoting its phosphorylation through Partitioning-defective 1 (PAR-1) and *Ca^2+^/calmodulin-dependent protein kinase* II (CaMKII), two known regulators of Dlg postsynaptic targeting (Koh et al., 1999; Wang et al., 2011; Zhang et al., 2007). Dlg is an important regulator of synaptic plasticity, and likely constitutes a major route by which Hts controls NMJ development (Ataman et al., 2006; Wang et al., 2011). In this study, we find that the main isoforms of Hts at the NMJ are the MARCKS-homology domain-containing isoforms, Add1 and/or Add2. There, the adducin-like isoforms form complexes with Dlg and PIP_2_, interactions that were identified through Proximity Ligation Assay (PLA). We provide evidence that Hts promotes the phosphorylation, and thus delocalization, of Dlg at the postsynaptic membrane by regulating the re-distribution of *par-1* and *camkII* transcripts from the muscle nucleus to the cytoplasm. We also show that these Hts interactions with Dlg and PIP_2_ are impeded through phosphorylation of the MARCKS-homology domain, further establishing that Hts is a signaling-responsive regulator of synaptic plasticity in *Drosophila*.

## RESULTS

### Add1 and/or Add2 are the predominant Hts isoforms present at the postsynaptic membrane of larval NMJs

**Fig. 1.**
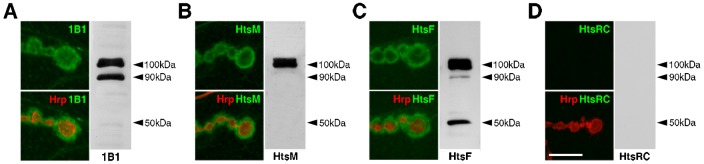
Hts isoforms present during larval NMJ development. (A–D) Comparative Western blot analyses of wild-type third instar larval body wall lysates blotted with Hts antibodies. Also shown are high magnification views of a few boutons from wild-type third instar larval NMJs at muscles 6/7 in abdominal segment 4, immunostained with Hts antibodies (green). Hrp is used to mark the neuronal membrane (red). (A) Blotting with the 1B1 antibody, which detects all Hts isoforms except for ShAdd, reveals a ∼100 kDa doublet representing Add1 and/or Add2, and a ∼90 kDa band that may represent either Hts-PD or Ovhts-Fus. NMJs show immunoreactivity predominantly at the postsynaptic membrane. (B) Blotting with the HtsM antibody, which detects only the adducin-like isoforms, reveals the same ∼100 kDa doublet found in the 1B1 blot. NMJs also show immunoreactivity predominantly at the postsynaptic membrane. (C) Blotting with the HtsF antibody, which detects all Hts isoforms, reveals the same bands found in the 1B1 blot, in addition to a ∼50 kDa band that may represent ShAdd. NMJs show a similar immunoreactivity pattern as observed with the 1B1 and HtsM antibodies. (D) Blotting with the HtsRC antibody, which detects only the Ovhts isoform, reveals no bands. NMJs also show no observable immunoreactivity. Scale bar in D represents 10 µm (for A–D).

We and another group have independently shown that the MARCKS-homology domain-containing isoforms of Hts, Add1 and/or Add2, are found at the *Drosophila* larval NMJ (Pielage et al., 2011; Wang et al., 2011). To determine if other Hts isoforms (i.e. ShAdd, Ovhts and Hts-PD) are present during larval NMJ development, we immunostained wild-type body walls with Hts isoform-specific antibodies (described in Petrella et al., 2007). As previously reported, when using the 1B1 antibody that detects all Hts isoforms except for ShAdd, Hts immunoreactivity is found predominantly at the postsynaptic membrane of type Ib and Is boutons, with lower levels found throughout the muscle (Fig. 1A) (Petrella et al., 2007; Pielage et al., 2011; Wang et al., 2011; Zaccai and Lipshitz, 1996). Compared to the 1B1 antibody, similar immunoreactivity patterns at the NMJ were observed when using the HtsF antibody that detects all Hts isoforms, as well as the HtsM antibody that detects the MARCKS-homology domain of the Add1 and Add2 isoforms exclusively (Fig. 1B,C) (Lin et al., 1994; Petrella et al., 2007; Pielage et al., 2011; Robinson et al., 1994; Wang et al., 2011). These results suggest that the adducin-like isoforms of Hts are the predominant isoforms present at the NMJ. In support of this, no detectable immunoreactivity was observed at the NMJ when using the HtsRC antibody that detects the RC domain of the Ovhts isoform, an expected result as Ovhts is restricted to the female germ line (Fig. 1D) (Petrella et al., 2007; Robinson et al., 1994; Telonis-Scott et al., 2009).

Western blot analyses of wild-type third instar larval body wall lysates revealed a ∼100 kDa doublet consistent with the observed sizes of Add1 and/or Add2 when blotting with the 1B1 antibody (Fig. 1A) (Petrella et al., 2007). We confirmed that the bands represented MARCKS-homology domain-containing Hts isoforms as blotting with the HtsM antibody showed a similar doublet (Fig. 1B). Blotting with the 1B1 antibody also revealed a smaller band at ∼90 kDa (Fig. 1A). Based on its size, the band may represent Hts-PD, a predicted 73.7 kDa isoform not previously detected *in vivo* but implicated in photoreceptor axon guidance via rescue assays (Ohler et al., 2011). Alternatively, the band may instead represent Ovhts-Fus, a ∼80 kDa posttranslational cleavage product of Ovhts found during oogenesis, as blotting adult ovary lysates with the 1B1 antibody shows a similar band corresponding to Ovhts-Fus just below the Add1/Add2 doublet (Petrella et al., 2007). The presence of Ovhts-Fus in our body wall lysates may be due to contaminating female germline tissue. However, blotting the body wall lysates with the HtsRC antibody revealed no Hts-specific immunoreactive bands, i.e. we did not observe a ∼60 kDa doublet corresponding to Ovhts-RC, the other Ovhts cleavage product, which is found in adult ovary lysates (Fig. 1D) (Petrella et al., 2007). To determine whether ShAdd, which is found in third instar larval brains, is also present during larval NMJ development, we used the HtsF antibody to check for additional bands not detected with the 1B1 antibody (Ohler et al., 2011). Indeed, the blot contained an additional band at ∼50 kDa that may represent ShAdd (Fig. 1C). Based on our Western blot analyses of larval body wall lysates, the adducin-like isoforms of Hts are prominently expressed. Other Hts isoforms appear to be also present, though we are not certain if they are found at the NMJ or in other cell types in the body wall.

### Hts postsynaptic targeting is disrupted through phosphorylation of the MARCKS-homology domain

Our immunohistological data indicate that Add1 and/or Add2 are the main Hts isoforms present at the postsynaptic membrane of larval NMJs. In mammalian adducins, the MARCKS-homology domain contains a conserved PKC/PKA target site, which when phosphorylated inhibits adducin's ability to regulate actin and spectrin (Ling et al., 1986; Matsuoka et al., 1996; Matsuoka et al., 1998; Waseem and Palfrey, 1988). To study the potential effects of Hts phosphorylation at the MARCKS-homology domain during larval NMJ development, we created non-phosphorylatable and phospho-mimetic *hts* transgenes. Single amino acid substitutions were made to the putative phosphorylation site in the MARCKS-homology domain of Hts by altering the codon sequence of cDNA encoding for the Add1 isoform through site-directed mutagenesis. The non-phosphorylatable *hts* transgene, *UAS-hts^S704A^*, contains a substitution of serine 704 to an alanine, a non-phosphorylatable amino acid. In contrast, the phospho-mimetic *hts* transgene, *UAS-hts^S704D^*, contains a substitution of the serine to an aspartic acid, i.e. a negatively charged amino acid that mimics a phosphorylative state. A wild-type *hts* transgene, *UAS-hts^S704S^*, was also made to serve as a control. Through Western blot analysis, we determined that all three transgenes had comparable expression levels, thus allowing us to perform direct comparative analyses (supplementary material Fig. S1).

**Fig. 2.**
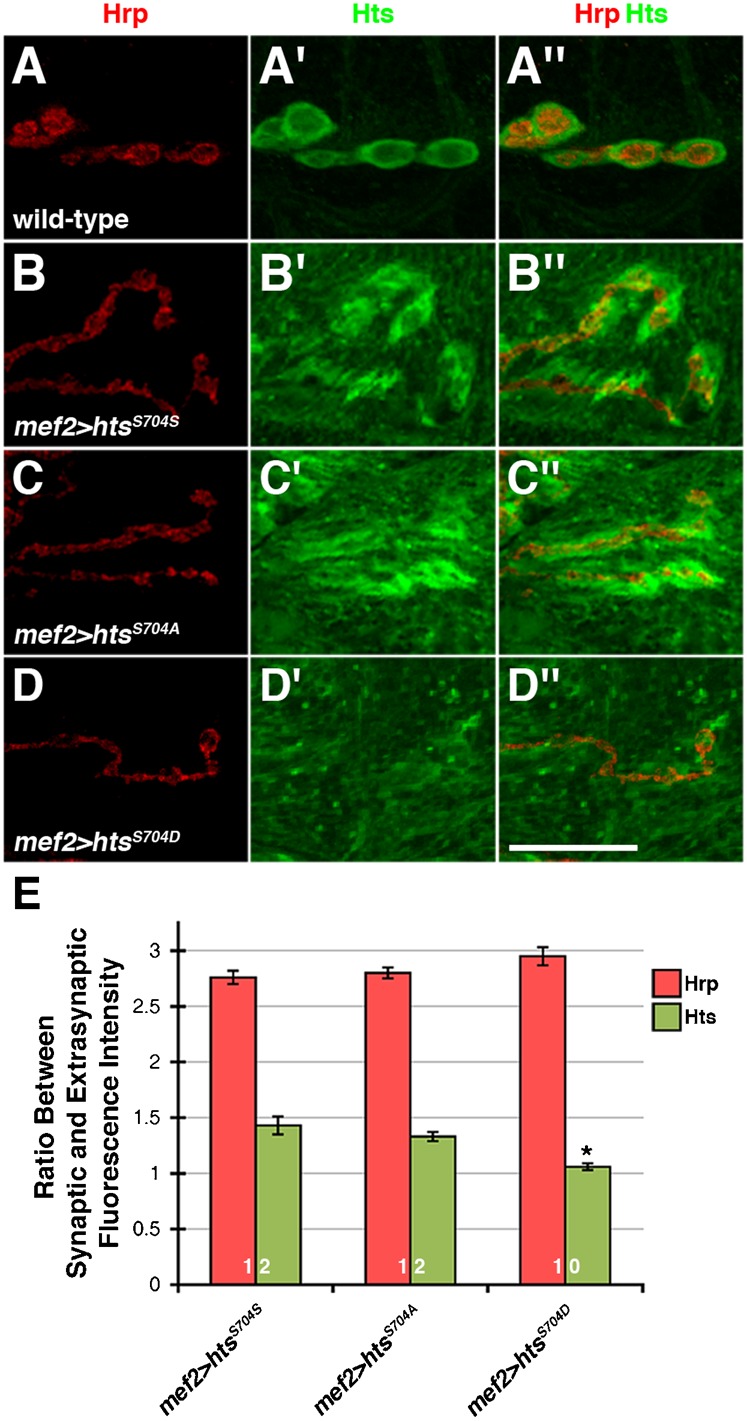
Phosphorylation of the MARCKS-homology domain disrupts Hts localization at the postsynaptic membrane of larval NMJs. (A–D″) High magnification views of a few boutons from third instar larval NMJs at muscles 6/7 in abdominal segment 4, immunostained with anti-Hrp (red) and 1B1 (green). (A–A″) Wild-type boutons showing endogenous, postsynaptic Hts distribution. (B–B″) Wild-type Hts, Hts^S704S^, expressed in the muscle with *mef2-Gal4* concentrates at the postsynaptic membrane around the boutons. (C–C″) Non-phosphorylatable Hts, Hts^S704A^, also concentrates at the postsynaptic membrane. (D–D″) In contrast to wild-type and non-phosphorylatable Hts, phospho-mimetic Hts, Hts^S704D^, does not specifically concentrate at the postsynaptic membrane. (E) Measurement of the ratio between synaptic and extrasynaptic Hts immunofluorescence intensity to quantitate Hts localization at the NMJ. The ratio for phospho-mimetic Hts is significantly lower in comparison to wild-type Hts, indicating that phospho-mimetic Hts does not localize as well to the NMJ. The numbers on the bars of the graph indicate the number of NMJs evaluated for each genotype. As both NMJs in abdominal segment 4 of each body wall were examined, the number of larvae evaluated for each genotype is half. Hrp was used as an internal control. p values were determined using one-way ANOVA analyses followed by Tukey-Kramer post hoc tests. *p < 0.01. Scale bar in Panel D″ represents 10 µm (for A–D″).

PKC-dependent phosphorylation causes adducin to translocate from the actin-spectrin cytoskeleton at cell-cell adhesion sites to the cytoplasm in epithelial cells (Matsuoka et al., 1998). Moreover, adducin is phosphorylated by PKC at elevated levels in renal carcinomas, and this aberrant phosphorylation correlates with changes in adducin subcellular distribution (Fowler et al., 1998). We assessed whether phosphorylation of the MARCKS-homology domain also regulates Hts localization at the postsynaptic membrane of larval NMJs. This was done by examining the distribution of wild-type, non-phosphorylatable and phospho-mimetic Hts when expressed in the muscle with *mef2-Gal4*. Both wild-type and non-phosphorylatable Hts concentrated at the postsynaptic membrane of NMJs (Fig. 2B–C″). Measurement of the ratio between synaptic and extrasynaptic Hts immunofluorescence intensity, which allowed us to quantitate Hts localization at the NMJ, revealed no significant difference between the two versions of Hts (Fig. 2E). However, phospho-mimetic Hts levels at the postsynaptic membrane were significantly reduced in comparison to wild-type and non-phosphorylatable Hts (Fig. 2D–E). The ratio was around 1, indicating that phospho-mimetic Hts does not specifically concentrate at the NMJ. These results show that phosphorylation of the MARCKS-homology domain disrupts Hts localization at the postsynaptic membrane of larval NMJs.

### Hts is in a complex with Dlg at the postsynaptic membrane of larval NMJs, and its regulation of Dlg localization is suppressed through phosphorylation of the MARCKS-homology domain

**Fig. 3.**
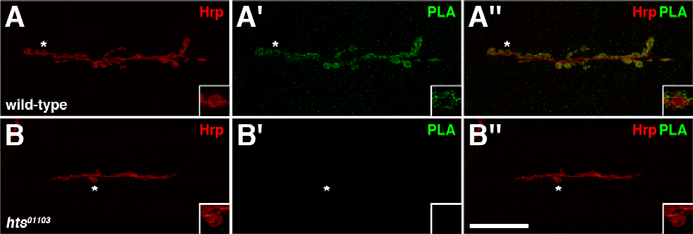
Hts and Dlg are in a complex at the postsynaptic membrane of larval NMJs. (A–B″) PLA with HtsM and Dlg antibodies (green) performed on third instar larval NMJs at muscles 6/7 in abdominal segment 4. Hrp is used to mark the neuronal membrane (red). Boutons marked with an asterisk (*) are shown at higher magnification in insets. (A–A″) Wild-type NMJs show punctate PLA signal between the adducin-like isoforms of Hts and Dlg at the postsynaptic membrane. (B–B″) *hts^01103^* mutant NMJs, which lack Hts immunoreactivity (not shown), exhibit no detectable PLA signal. Scale bar in Panel B″ represents 20 µm (for A–B″).

We previously showed that Hts and Dlg form a complex based on co-immunoprecipitation experiments done on whole adult fly lysates (Wang et al., 2011). One shortcoming of these results, however, is that they do not tell us where this complex forms. With immunohistochemistry, the distributions of Hts and Dlg are observed to overlap at the postsynaptic membrane of larval NMJs, but are they in a complex in this region (Wang et al., 2011)? To address this question, we used PLA to look for an *in situ* association between endogenous Hts and Dlg at the NMJ (Söderberg et al., 2006). In this assay, which to our knowledge has not been used to study the *Drosophila* larval NMJ before, wild-type body walls were immunostained with HtsM and Dlg primary antibodies, where the Dlg antibody recognizes the PDZ domains at the N terminus (Parnas et al., 2001). The primary antibodies were then detected with species-specific secondary antibodies, termed PLA probes, which are conjugated to oligonucleotides. If Hts and Dlg are in close proximity to each other (i.e. within a few tens of nanometers), the distance between the attached PLA probes can be bridged through hybridization of two additional connector oligonucleotides (Söderberg et al., 2006). In this conformation, the free ends of the connector oligonucleotides can make contact with each other, and a closed circular DNA molecule is formed upon *in situ* ligation. The circular DNA molecule serves as a template for *in situ* rolling circle amplification, which is primed by one of the PLA probes. Sequences within the amplified, concatemeric DNA product can then be visualized with fluorescently-labeled, complementary oligonucleotide probes. Since the amplified DNA remains attached to one of the PLA probes, the subcellular location of the interaction can be ascertained. In our PLA experiments, wild-type NMJs showed punctate signal between the adducin-like isoforms of Hts and Dlg at the postsynaptic membrane, thus indicating that these proteins are in close proximity to each other in this region and likely form a comple*x* (Fig. 3A–A″). The signal was determined to be specific as *hts^01103^* mutant NMJs, which have been shown to lack Hts immunoreactivity, displayed no observable signal (Fig. 3B–B″) (Wang et al., 2011). Additionally, PLA performed without adding the HtsM antibody also resulted in no signal (data not shown).

**Fig. 4.**
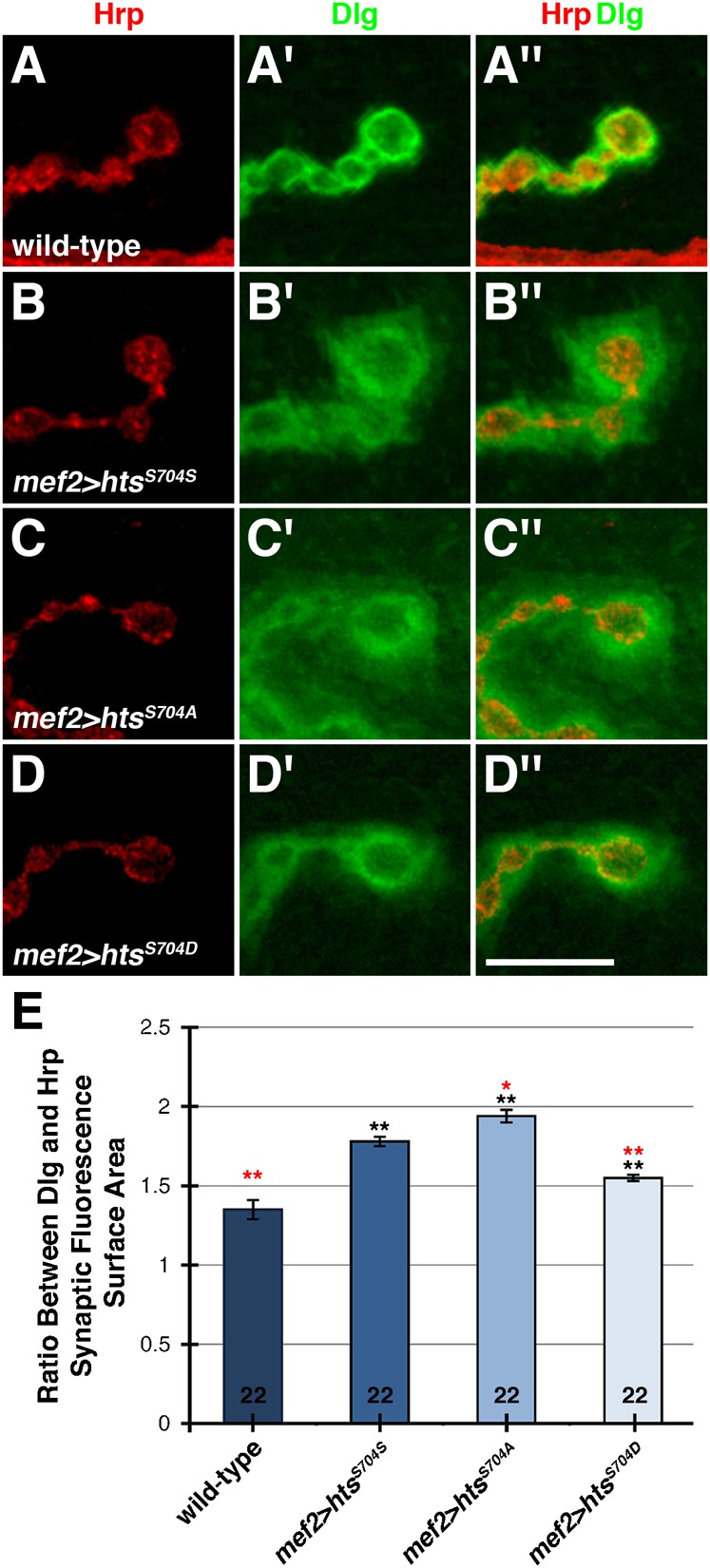
Phosphorylation of the MARCKS-homology domain partially inhibits Hts' ability to disrupt Dlg postsynaptic targeting to the larval NMJ. (A–D″) High magnification views of a few boutons from third instar larval NMJs at muscles 6/7 in abdominal segment 4, immunostained with anti-Hrp (red) and anti-Dlg (green). (A–A″) Wild-type boutons show a tight association of Dlg to the postsynaptic membrane. (B–B″) Wild-type Hts expressed in the muscle with *mef2-Gal4* disrupts Dlg postsynaptic targeting, as Dlg distribution appears diffuse and extends farther away from the NMJ. (C–C″) Non-phosphorylatable Hts disrupts Dlg postsynaptic targeting to a greater degree when compared to wild-type Hts, as Dlg distribution appears more diffuse and Dlg levels in the surrounding muscle are higher. (D–D″) In comparison to wild-type and non-phosphorylatable Hts, phospho-mimetic Hts disrupts Dlg postsynaptic targeting to a lesser degree, as Dlg distribution appears less diffuse. (E) Measurement of the ratio between Dlg and Hrp immunofluorescence surface area at the NMJ to quantitate the extent of Dlg spreading from the presynaptic membrane. The ratio for non-phosphorylatable Hts is significantly higher in comparison to wild-type Hts, indicating that blocking Hts phosphorylation enhances disruption of Dlg postsynaptic targeting. In contrast, the ratio for phospho-mimetic Hts is significantly lower when compared to wild-type Hts, indicating that Hts phosphorylation reduces disruption of Dlg postsynaptic targeting. The numbers on the bars of the graph indicate the number of NMJs evaluated for each genotype. As both NMJs in abdominal segment 4 of each body wall were examined, the number of larvae evaluated for each genotype is half. p values were determined using one-way ANOVA analyses followed by Tukey-Kramer post hoc tests. Asterisks in black show significance compared to the wild-type control. Asterisks in red show significance compared to transgenic wild-type Hts expression. *p < 0.05; **p < 0.01. Scale bar in Panel D″ represents 10 µm (for A–D″).

We were interested to see if Hts-mediated regulation of Dlg postsynaptic targeting was controlled through phosphorylation of the MARCKS-homology domain. We evaluated the localization of Dlg at the postsynaptic membrane following expression of the wild-type, non-phosphorylatable and phospho-mimetic *hts* transgenes in the muscle with *mef2-Gal4*. With postsynaptic expression of the Gene Search line, *GS13858*, which results in elevated levels of all Hts isoforms, we previously showed that Hts negatively regulates Dlg localization (Wang et al., 2011). Over-expression of only the Add1 isoform using our wild-type *hts* transgene also resulted in the disruption of Dlg postsynaptic targeting, as Dlg localization around the NMJ appeared diffuse in comparison to a wild-type control (Fig. 4A–B″). This result indicates that the MARCKS-homology domain-containing Hts isoforms are responsible for regulating the localization of Dlg at the postsynaptic membrane of larval NMJs. In support of this, expression of transgenes encoding for ShAdd and Ovhts did not affect Dlg localization (data not shown). Interestingly, non-phosphorylatable Hts expression seemed to disrupt Dlg postsynaptic targeting more severely than wild-type Hts expression, as Dlg localization around the NMJ appeared more diffuse and Dlg levels in the surrounding muscle appeared higher (Fig. 4C–C″). Phospho-mimetic Hts expression also disrupted Dlg postsynaptic targeting, however, Dlg localization appeared less diffuse around the NMJ when compared to the expression of wild-type and non-phosphorylatable Hts (Fig. 4D–D″). These results were quantified by determining the ratio between Dlg and Hrp immunofluorescence surface area at the NMJ, a measurement that allowed us to assess the extent of Dlg ‘spreading away’ from the presynaptic membrane (Fig. 4E). The ratio for non-phosphorylatable Hts was significantly higher when compared to wild-type Hts, indicating that blocking phosphorylation of Hts enhances disruption of Dlg postsynaptic targeting. In contrast, the ratio for phospho-mimetic Hts was significantly lower in comparison to wild-type Hts, indicating that phosphorylation of Hts reduces disruption of Dlg postsynaptic targeting. Thus, we provide evidence that phosphorylation of Hts in the MARCKS-homology domain suppresses its ability to regulate Dlg localization at the postsynaptic membrane during larval NMJ development.

### Hts regulates *par-1* and *camkII* mRNA distribution and levels in the muscle

We considered whether Hts ‘pulls’ Dlg away from the postsynaptic membrane, given their close association. However, co-localization analysis between the ectopic distribution of over-expressed wild-type Hts and the resulting diffuse distribution of Dlg showed that the distributions did not exactly match (supplementary material Fig. S2), thus arguing against Hts simply ‘pulling’ Dlg away from the NMJ. In support of this, we have shown before that elevated Hts protein in embryonic epithelia, which accumulates at the cell membrane, results in Dlg delocalization away from the membrane (Wang et al., 2011). These results indicate that other processes must contribute to Hts-mediated regulation of Dlg localization.

**Fig. 5.**
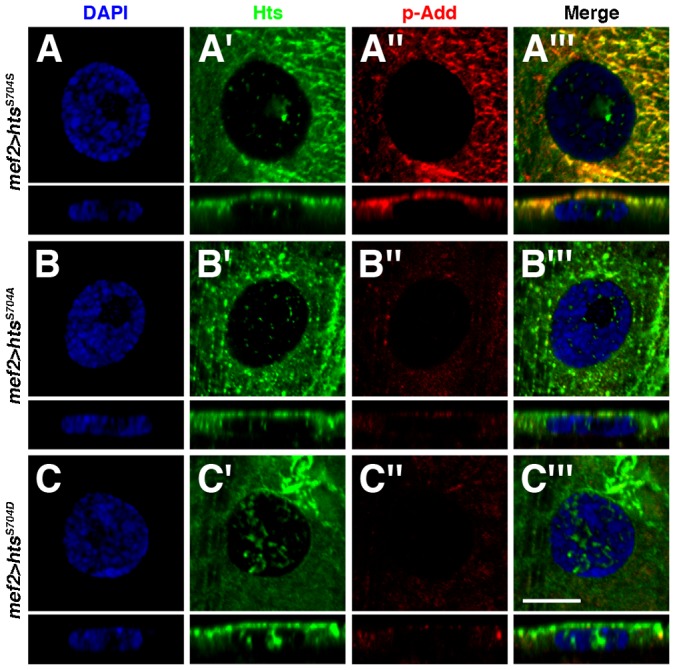
Transgenic Hts localizes in muscle nuclei. (A–C′″) High magnification views of nuclei (single sections taken within the nucleus) in muscles 6 or 7 in abdominal segment 4, stained with 1B1 (green), anti-phospho-adducin (red) and the DAPI nuclear marker (blue). Bottom panels show cross-sectional views. (A–A′″) When over-expressing wild-type Hts in the muscle with *mef2-Gal4*, Hts immunoreactivity is observed in the nucleus as discreet puncta, with accumulations in the nucleolus sometimes observed. However, phosphorylated Hts, detected with an antibody against adducin phosphorylation at the conserved site in the MARCKS-homology domain, is not observed. Note that the immunoreactivities of Hts and phospho-adducin overlap outside of the nucleus. (B–B′″) Non-phosphorylatable Hts shows a similar nuclear distribution pattern as wild-type Hts. (C–C′″) Surprisingly, phospho-mimetic Hts is also observed in the nucleus, though it forms abberant accumulations that differ from the discreet puncta seen with wild-type and non-phosphorylatable Hts. Note that unlike with wild-type Hts expression, elevations in phospho-adducin levels in the muscle are not detected by the phospho-adducin antibody when expressing the non-phosphorylatable and phospho-mimetic *hts* transgenes as they contain alterations of the target serine residue. Scale bar in Panel C′″ represents 10 µm (for A–C′″).

Prior studies have shown that Dlg localization at the postsynaptic membrane of larval NMJs is disrupted through phosphorylation by either PAR-1 or CaMKII (Koh et al., 1999; Zhang et al., 2007). We previously provided evidence that postsynaptic over-expression of Hts, using *GS13858*, results in elevated PAR-1 and CaMKII protein levels throughout the muscle, thereby promoting Dlg phosphorylation and delocalization (Wang et al., 2011). Interestingly, mammalian α-adducin can shuttle between cell-cell junctions and the nucleus in epithelia due to the presence of a bipartite nuclear localization signal (NLS) in the MARCKS-homology domain and a nuclear export signal (NES) in the neck region (Chen et al., 2011). In the nucleus, α-adducin is required for proper spindle assembly and mitotic progression (Chan et al., 2014; Chen et al., 2011). Upon determining that both the NLS and NES sequences are conserved in Hts, we wondered if the *Drosophila* adducins are present in muscle nuclei where they can affect other processes such as gene expression. In wild-type body wall muscles, Hts nuclear immunoreactivity was not readily detectable (data not shown). However, when wild-type Hts was over-expressed in the muscle, Hts immunoreactivity was observed in nuclei as discreet puncta (Fig. 5A′).

**Fig. 6.**
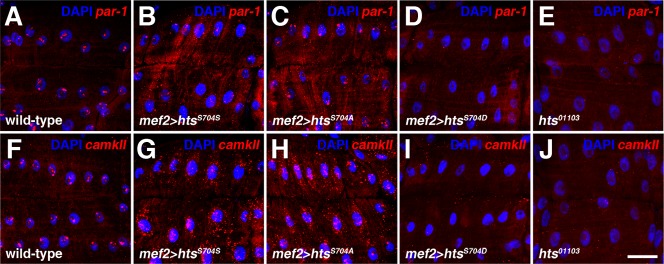
Hts regulates *par-1* and *camkII* mRNA distribution and levels in the muscle. (A–J) Views of muscles 6/7 in abdominal segment 4, probed for either *par-1* or *camkII* mRNA (red). Nuclei are marked with DAPI (blue). (A,F) In wild-type larval body walls, *par-1* and *camkII* mRNA are predominately sequestered in muscle nuclei. (B,C,G,H) In contrast, expression of either wild-type or non-phosphorylatable Hts with *mef2-Gal4* causes *par-1* and *camkII* mRNA, observed as distinct puncta, to be dispersed throughout the muscle cytoplasm. (D,I) Expression of phospho-mimetic Hts, on the other hand, results in overall reductions in *par-1* and *camkII* mRNA levels in both the muscle nuclei and cytoplasm. (E,J) *hts^01103^* mutant larval body walls also show reduced *par-1* and *camkII* mRNA levels. Scale bar in Panel J represents 20 µm (for A–J).

To address whether Hts regulates PAR-1 and/or CaMKII at the transcript level during larval NMJ development, we performed fluorescent *in situ* hybridisation (FISH). In wild-type body walls, *par-1* and *camkII* mRNA were predominantly sequestered in muscle nuclei as distinct accumulations (Fig. 6A,F). In striking contrast, muscle-specific expression of either wild-type or non-phosphorylatable Hts caused *par-1* and *camkII* mRNA, which were observed as discreet puncta, to be dispersed throughout the muscle cytoplasm (Fig. 6B,C,G,H). These results show that Hts can promote the export of transcripts from muscle nuclei into the cytoplasm. The antisense probes were determined to be specific as expression of transgenic RNAi against the transcript of interest abolished FISH signal (supplementary material Fig. S3B,F), while over-expression of the transcript of interest elevated FISH signal (supplementary material Fig. S3C,G). Experiments were also performed with sense probes that resulted in no FISH signal, thus further validating our observed results (supplementary material Fig. S3D,H).

When expressing phospho-mimetic Hts in the muscle, overall *par-1* and *camkII* mRNA levels in the nuclei and cytoplasm were reduced, a result also observed in *hts^01103^* mutants (Fig. 6D,E,I,J). These results show that Hts is required for the transcription and/or stability of *par-1* and *camkII* transcripts, in addition to their nuclear export, and that these functions are impeded by Hts phosphorylation in the MARCKS-homology domain. Moreover, they indicate that phospho-mimetic Hts displays a dominant negative effect. Interestingly, phosphorylated Hts, which was detected with an antibody against adducin phosphorylation at the MARCKS-homology domain, was not observed in nuclei when wild-type Hts was over-expressed in the muscle (Fig. 5A″). The antibody has previously been determined to be specific to Hts (Pielage et al., 2011). Based on the phospho-adducin distribution, we expected phospho-mimetic Hts to be absent from muscle nuclei, thereby providing a possible explanation as to its inability to positively regulate *par-1* and *camkII* transcript levels in the muscle cytoplasm. However, phospho-mimetic Hts was clearly observed in nuclei when expressed in the muscle, though it formed abberant accumulations that differed from the distinct puncta seen with wild-type and non-phosphorylatable Hts (Fig. 5A–C′″). One possible explanation for the discrepancy in the nuclear localizations between endogenous phosphorylated Hts and phospho-mimetic Hts is that the phosphorylated serine residue may be important for Hts' exclusion from the nucleus.

### Hts interacts with PIP_2_ via the MARCKS-homology domain at the postsynaptic membrane of larval NMJs

We have shown before that muscle-specific elevations in Hts result in overdeveloped larval NMJs with significant increases in branch number and length (Wang et al., 2011). This phenotype cannot solely be due to observed increases in PAR-1 and CaMKII that lead to Dlg delocalization, as other studies have shown that over-expression of either PAR-1 or constitutively active CaMKII in the muscle causes a marked reduction in NMJ branching complexity (Koh et al., 1999; Zhang et al., 2007). Thus, Hts is likely acting through additional synaptic components to regulate larval NMJ development. One potential candidate is PIP_2_, a plasma membrane lipid that acts as a substrate for multiple signaling pathways. In *Drosophila*, presynaptic PIP_2_ plays a role in restricting NMJ growth through regulation of WASP signaling (Khuong et al., 2010).

**Fig. 7.**
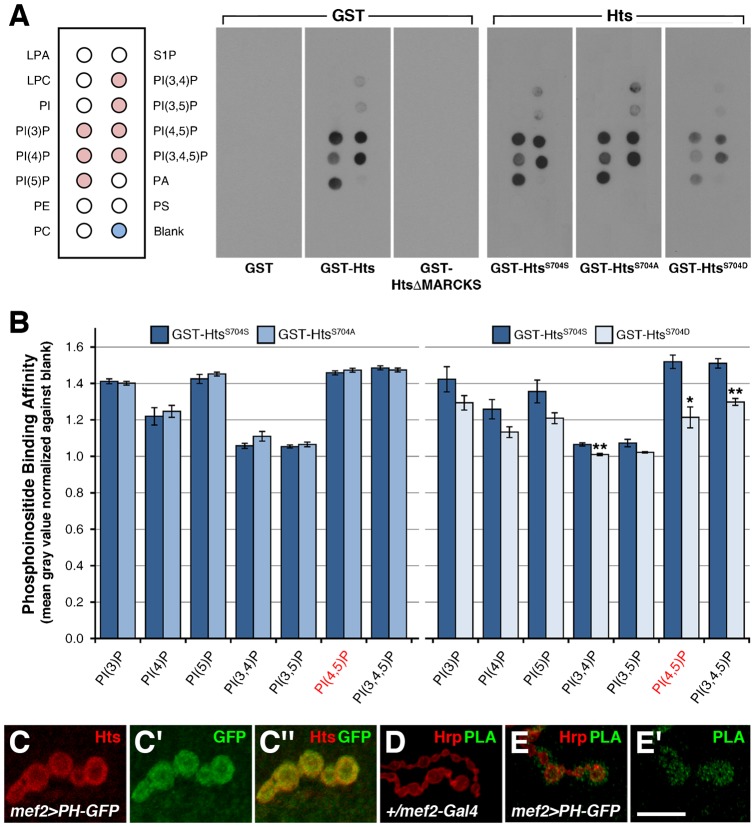
Hts binds to phosphoinositides and is in a complex with PIP_2_ at the postsynaptic membrane of larval NMJs. (A) Binding of various GST-Hts constructs on PIP strips. GST-Hts binds to all phosphoinositides (see pink dots on schematic for reference), including a strong affinity for PIP_2_ (PI(4,5)P). GST alone and GST-HtsΔMARCKS do not bind to any of the lipids. Phospho-mimetic GST-Hts^S704D^ binds with less affinity to multiple phosphoinositides in comparison to wild-type GST-Hts^S704S^. No significant differences between non-phosphorylatable GST-Hts^S704A^ and GST-Hts^S704S^ are observed, indicating that alteration of the serine residue is not responsible for the observed changes in binding affinities. (B) Quantification of binding affinities through measuring the signal strengths from each lipid spot. GST-Hts^S704D^ binding to certain phosphoinositides, including PIP_2_, is significantly reduced in comparison to GST-Hts^S704S^. No significant differences between GST-Hts^S704A^ and GST-Hts^S704S^ are observed. Experiments were done in triplicate. p values were determined using pairwise Student's t-tests. *p < 0.01; **p < 0.005. (C–C″) High magnification view of a few boutons from a third instar larval NMJ at muscles 6/7 in abdominal segment 4, immunostained with anti-HtsM (red) and anti-GFP (green). PH-GFP, a known binder of PIP_2_, is expressed in the muscle with *mef2-Gal4*. PH-GFP concentrates specifically around the postsynaptic membrane where it overlaps with the adducin-like isoforms of Hts. (D–E′) PLA with HtsM and GFP antibodies (green) performed on third instar larval NMJs at muscles 6/7 in abdominal segment 4. Hrp is used to mark the neuronal membrane (red). (D) *mef2-Gal4* alone does not show any observable PLA signal. (E–E′) Expressing *UAS-PH-GFP* in the muscle with *mef2-Gal4* results in PLA signal at the postsynaptic membrane. Scale bar in Panel E′ represents 10 µm (for C–E′).

We used PIP strips to determine if the adducin-like isoforms of Hts can bind to PIP_2_. GST fusion proteins were expressed in bacteria, and then purified with Glutathione Sepharose (supplementary material Fig. S4). GST-Hts (1–717 aa), consisting of full length Add1, showed binding to all phosphoinositides including a strong affinity for PIP_2_, whereas GST alone showed no binding at all (Fig. 7A). The interaction was determined to be dependent on the MARCKS-homology domain as GST-HtsΔMARCKS (1–687 aa) did not bind to any of the lipids (Fig. 7A). In support of this result, GST-MARCKS (688–717 aa) displayed a similar binding pattern as GST-Hts (data not shown). We were curious to see whether phosphorylation of the MARCKS-homology domain affected phosphoinositide binding. Indeed, phospho-mimetic GST-Hts^S704D^ bound with less affinity to multiple phosphoinositides when compared to wild-type GST-Hts^S704S^ (Fig. 7A). This was confirmed by measuring the signal strength of each lipid spot, which showed that phospho-mimetic GST-Hts^S704D^ binding to certain phosphoinositides, including PIP_2_, was significantly reduced in comparison to wild-type GST-Hts^S704S^ (Fig. 7B). No significant differences were observed between non-phosphorylatable GST-Hts^S704A^ and wild-type GST-Hts^S704S^, indicating that alteration of the serine residue was not responsible for the observed changes in binding affinities (Fig. 7A,B). These results show that Hts binds directly to PIP_2_ via the MARCKS-homology domain. Moreover, this interaction is partially inhibited through phosphorylation of the MARCKS-homology domain.

We next assessed whether the interaction between Hts and PIP_2_ exists at the postsynaptic membrane of larval NMJs. To visualize endogenous PIP_2_ distribution, we used a *UAS-PH-GFP* reporter that encodes for the pleckstrin homology (PH) domain of phospholipase C_δ1_, a known binder of PIP_2_ (Várnai and Balla, 1998; Verstreken et al., 2009). When expressed in the muscle, PH-GFP concentrated specifically around the postsynaptic membrane where it largely overlapped with Hts (Fig. 7C–C″). Furthermore, Hts and PH-GFP were determined to be in a complex through PLA involving the HtsM and GFP antibodies. In NMJs where *UAS-PH-GFP* was expressed in the muscle with *mef2-Gal4*, PLA signal was observed at the postsynaptic membrane, whereas *mef2-Gal4* alone did not show any observable signal (Fig. 7D,E). Since the HtsM antibody was used in these assays, it is the MARCKS-homology domain-containing isoforms of Hts that form a complex with PH-GFP, and thus PIP_2_, at the postsynaptic membrane of larval NMJs.

## DISCUSSION

### Hts is in a complex with Dlg and PIP_2_ at the NMJ where it regulates synaptic development

**Fig. 8.**
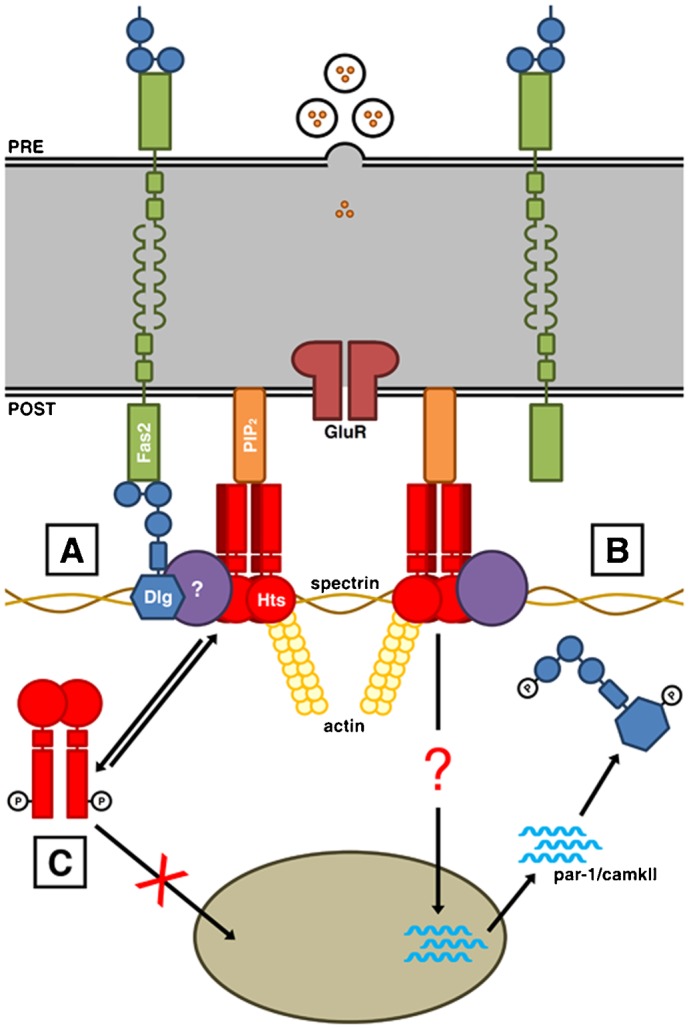
Phosphorylation of the MARCKS-homology domain regulates Hts' interactions with Dlg and PIP_2_ at the postsynaptic membrane of larval NMJs. Schematic of a larval NMJ viewed in cross-section, with the presynaptic side shown at the top and the postsynaptic side shown at the bottom. (A) At the postsynaptic membrane, Dlg localizes to spectrin-actin complexes. Homophilic adhesion between Fasciclin 2 (Fas2) transmembrane proteins links the presynaptic and postsynaptic sides, with the intracellular C-terminal domains anchored to the first and second PDZ domains of Dlg. Hts is in a complex with Dlg at the postsynaptic membrane, though the interaction may not be direct. Hts also binds to PIP_2_ via the MARCKS-homology domain. (B) Hts promotes the accumulation of *par-1* and *camkII* transcripts in the muscle cytoplasm through an as of yet identified mechanism. PAR-1 and CaMKII phosphorylate Dlg at serine 797 in the GUK domain and serine 48 in one of the PDZ domains respectively. Phosphorylation at either one of these sites disrupts Dlg postsynaptic targeting. (C) Phosphorylation of serine 704 in the MARCKS-homology domain translocates Hts away from the postsynaptic membrane and hinders Hts' ability to regulate Dlg localization, presumably through the control of PAR-1 and CaMKII at the transcriptional level. Phosphorylation of the MARCKS-homology domain also inhibits Hts' ability to bind to PIP_2_.

Through the use of PLA, we show here that Hts forms complexes with Dlg and PIP_2_ at the postsynaptic region of the larval NMJ, with its ability to associate with these proteins being negatively regulated through phosphorylation of the MARCKS-homology domain (Fig. 8). Studies on mammalian adducin have demonstrated that phosphorylation of the MARCKS-homology domain impedes its actin-binding and spectrin-recruiting functions, reduces its affinity for these cytoskeletal components and the membrane, and targets it for proteolysis (Barkalow et al., 2003; Gilligan et al., 2002; Matsuoka et al., 1996; Matsuoka et al., 1998; Pariser et al., 2005). We propose that phosphorylation of the MARCKS-homology domain in the Add1/Add2 isoforms of Hts in response to upstream signaling events at the synapse reduces their affinity for spectrin-actin junctions and Dlg at the NMJ, but may also hinder their interactions with PIP_2_ and other phosphoinositides in line with the electrostatic switch model for phosphoinositide binding by the MARCKS-homology domain (Golebiewska et al., 2006; McLaughlin and Aderem, 1995).

We have previously proposed that Hts regulates Dlg localization at the NMJ by controlling the protein levels of PAR-1 and CaMKII, which phosphorylate Dlg and disrupt its postsynaptic targeting (Koh et al., 1999; Wang et al., 2011; Zhang et al., 2007). We now show that regulation of these kinases appears to occur at the level of transcript processing, with Hts promoting the accumulation of *par-1* and *camkII* transcripts in the muscle cytoplasm (Fig. 8). Cytoplasmic accumulation of the transcripts would then presumably lead to higher levels of PAR-1 and CaMKII protein. How is Hts achieving this mode of regulation when it is residing with Dlg at the postsynaptic membrane? One possibility is that Hts at the NMJ is activating a signaling pathway that promotes the transcription and/or stability of *par-1* and *camkII* transcripts, as well as their transport out of the nucleus. Another possibility is that Hts itself, which contains predicted NLS and NES sequences, translocates to the nucleus in response to events at the NMJ, similar to the way that mammalian α-adducin translocates to the nucleus upon loss of cell-cell adhesion in epithelia (Chen et al., 2011). We have been unable to detect endogenous Hts in muscle nuclei, however, nuclear Hts levels might be tightly restricted and undetectable under wild-type conditions. Over-expressed wild-type Hts, on the other hand, is readily observable in the nucleus, though not its phosphorylated form – a result also seen with α-adducin (Chen et al., 2011). Whatever the mechanism may be, the presence of Hts in a complex with Dlg may allow it to evaluate the status of Dlg and the synapse, and execute a response in the form of regulating Dlg localization through PAR-1 and CaMKII mediated phosphorylation.

A recent study has uncovered a novel nuclear envelope budding mechanism that can export select transcripts from muscle nuclei during larval NMJ development, and involves Lamin C (LamC) and the Wnt receptor, DFrizzled2 (DFzz2) (Speese et al., 2012). Interestingly, *camkII*, but not *dlg*, transcripts are regulated by this process, which is consistent with our findings that CaMKII, but not Dlg, expression is regulated by Hts (this study and Wang et al., 2011). Future work will determine whether Hts is involved in this LamC/DFzz2-dependent mechanism.

Two papers have underscored the importance of phosphoinositides in synaptic development at the *Drosophila* NMJ (Forrest et al., 2013; Khuong et al., 2010). Binding of Hts to PIP_2_ and probably other phosphoinositides at the NMJ, as seen with other MARCKS-homology domain-containing proteins, may affect the availability of these lipids for processes such as signal transduction, thus affecting synaptic development. Conversely, the localization of Hts at the NMJ may be regulated by the distribution of phosphoinositides. In line with this, postsynaptic knockdown of the phosphoinositide phosphatase Sac1 via transgenic RNAi expression disrupts Hts localization at the NMJ (Forrest et al., 2013).

### Hts/adducin and synaptic function and dysfunction

The observations reported in this study may have important implications for understanding diseases that affect synaptic function in humans and other mammals. Many neurodegenerative diseases including amyotrophic lateral sclerosis (ALS), a disorder characterized by the progressive loss of motor neurons, have been assumed until recently to be a consequence of neuronal death within the central nervous system. However, there is substantial recent evidence indicating that neuron pathology in ALS and other neurodegenerative diseases is due to a degenerative process that begins in the presynaptic terminal, NMJ or distal axon (Fischer et al., 2004). This may also be the case in normal aging (Valdez et al., 2012).

We initially became interested in adducin when we found elevated levels of phospho-adducin protein in the spinal cord tissue of patients who died with ALS, compared to individuals who died without neurological disease (Hu et al., 2003). Similar observations were also made in mSOD-expressing mice, a transgenic animal model of ALS (Shan et al., 2005). Multiple studies have shown that adducin plays important roles in synaptic plasticity, and that mice mutant for β-adducin display defects in memory, learning and motor coordination (Porro et al., 2010). As is shown here and in other work, it is clear that modulation of Hts expression and phosphorylation can affect synaptic development (Bednarek and Caroni, 2011; Pielage et al., 2011; Wang et al., 2011). We provide evidence here that phosphorylation of Hts impedes its function at the larval NMJ, a result that is consistent with the mammalian adducins. In addition, overexpression of phospho-mimetic Hts has dominant negative effects over endogenous Hts. Thus, loss of adducin function through aberrant phosphorylation of the MARCKS-homology domain may be a contributing factor for human neurodegenerative diseases.

## MATERIALS AND METHODS

### Fly Stocks

Flies were maintained under standard conditions at 25°C (Ashburner, 1989). *w^1118^* was used as a wild-type control unless otherwise stated. *w^1118^*, *hts^01103^* (Spradling et al., 1999) and *mef2-Gal4* were from the Bloomington *Drosophila* Stock Center, while *UAS-par-1^RNAi^* and *UAS-camkII^RNAi^* were from the Vienna *Drosophila* RNAi Center (Dietzl et al., 2007). The following stocks were kindly provided by other labs: *UAS-par-1-GFP* (Huynh et al., 2001) from Dr. Daniel St Johnston, *UAS-camkII* (Jin et al., 1998) from Dr. Leslie Griffith and *UAS-PH-GFP* (Khuong et al., 2010) from Dr. Julie Brill.

### Transgenic Constructs

The SD02552 cDNA clone (Rubin et al., 2000), which encodes the Add1 isoform, was obtained from the *Drosophila* Genomics Resource Center. The full-length coding region was first subcloned into pBlueScript II SK(+). Mutations in the putative phosphorylation site of the MARCKS-homology domain at serine 704 were next created using the QuikChange II Site-Directed Mutagenesis Kit (200523 – Agilent Technologies). The following primers were used (only forward primers are shown), with the modified codons bolded: 5′-GAAGGGTCTGCGCACACCA**GCC**TTTTTGAAAAAGAAGAAGG-3′ (non-phosphorylatable) and 5′-GAAGGGTCTGCGCACACCA**GAC**TTTTTGAAAAAGAAGAAGG-3′ (phospho-mimetic). Wild-type, non-phosphorylatable and phospho-mimetic *hts* cDNA inserts were then subcloned into pUAST. P element transformation was performed by BestGene.

### Larval Body Wall Preparation

Body wall dissections of crawling third instar larvae were performed as previously described (Brent et al., 2009; Ramachandran and Budnik, 2010a). Mutant stocks were re-balanced over GFP-tagged balancers allowing for homozygotes to be selected based on the absence of GFP signal. For transgenic analysis, homozygous *UAS*-transgene-bearing males were crossed to homozygous *Gal4*-bearing virgin females ensuring that all progeny carried one copy of each.

### Immunohistochemistry

Immunostaining of body walls was performed as previously described (Ramachandran and Budnik, 2010b). The following primary antibodies were used: 1:500 rat anti-HtsF (Lin et al., 1994; Robinson et al., 1994) from Dr. Lynn Cooley, 1:5 mouse anti-Hts-1B1 (Zaccai and Lipshitz, 1996) (1B1 – Developmental Studies Hybridoma Bank), 1:200 rabbit anti-HtsM (Petrella et al., 2007) from Dr. Cooley, 1:10 mouse anti-HtsRC (Robinson et al., 1994) (hts RC – DSHB), 1:200 goat anti-Hrp (123-005-021 – Jackson ImmunoResearch), 1:10 mouse anti-Dlg (Parnas et al., 2001) (4F3 – DSHB), goat anti-phospho-adducin (sc-12614 – Santa Cruz Biotechnology) and 1:500 mouse anti-GFP (G1544 – Sigma-Aldrich). Fluorescent-labeled secondary antibodies from Vector Laboratories were used at a 1:200 dilution. Stained body walls were stored in VECTASHIELD Mounting Medium with DAPI (H-1200 – Vector Laboratories). Images of NMJs at muscles 6/7 from abdominal segment 4 were taken as merged stacks (unless otherwise stated) on a Nikon A1R laser scanning confocal microscope with NIS-Elements software, with experiments and their controls imaged under identical acquisition settings. All images were processed with Adobe Photoshop.

### Quantification

All quantifications were performed with Adobe Photoshop. Stacked confocal images were converted to grayscale, and their resolutions were standardized to 300 pixels/inch. Hts postsynaptic targeting was quantified by measuring the ratio between Hts immunofluorescence intensity at the NMJ versus the extrasynaptic region. Hts immunofluorescence signal at the NMJ was selected using the Color Range tool, and the selection was refined using the Smooth function under the Modify tool. Immunofluorescence intensity was then measured as mean gray value. The selection was next altered using the Border function under the Modify tool to select the extrasynaptic region, and immunofluorescence intensity was measured as mean gray value. Hrp was used as an internal control. Dlg postsynaptic targeting was quantified by measuring the ratio between Dlg and Hrp immunofluorescence surface area at the NMJ. Immunofluorescence signal at the NMJ was selected using the same procedure as described above, and the surface area was then measured as pixel area. Note that the parameters used for the quantifications were kept constant within data sets.

Measurements were performed on NMJs at muscles 6/7 from abdominal segment 4. Data were expressed as absolute values to the control and presented as ‘mean±sem’. One-way ANOVA analyses followed by Tukey-Kramer post hoc tests were performed for all statistical comparisons.

### Western Blot

Lysates were composed of 25 dissected body walls. For each sample, the protein concentration was determined using the Bio-Rad Protein Assay (500-0006 – Bio-Rad), and 20 µg of total soluble protein was loaded onto an 8% SDS-PAGE gel. The following primary antibodies were used for blotting: 1:7500 rat anti-HtsF, 1:1500 mouse anti-Hts-1B1, 1:5000 rabbit anti-HtsM, 1:100 mouse anti-HtsRC and 1:1000 mouse anti-Actin (JLA20 – DSHB). Peroxidase-conjugated secondary antibodies from Vector Laboratories were used at a 1:2000 dilution. Detection was accomplished with the BM Chemiluminescence Western Blotting Peroxidase Substrate (11500694001 – Roche Applied Science).

### Proximity Ligation Assay

PLA was performed as previously described but with some modifications (Thymiakou and Episkopou, 2011). Dissected body walls were blocked with 1% BSA (in PBT - 0.03M NaH_2_PO_4_, 0.07M Na_2_HPO_4_, 1.3M NaCl, 0.1% Triton X-100, pH 7.0) for one hour. The body walls were next incubated with rabbit and mouse primary antibodies against the two proteins of interest, along with a 1:200 dilution of goat anti-Hrp to mark neuronal membranes, in 1% BSA for two hours. The following primary antibodies were used: 1:200 rabbit anti-HtsM, 1:10 mouse anti-Dlg and 1:500 mouse anti-GFP. To detect the goat anti-Hrp antibody, the body walls were then incubated with a 1:200 dilution of a FITC-labeled anti-goat secondary antibody (705-095-003 – Jackson ImmunoResearch) in 1% BSA overnight at 4°C. After three washes with PBT for ten minutes each, the body walls were incubated with a 1:5 dilution of anti-rabbit PLUS (DUO92002 – Sigma-Aldrich) and anti-mouse MINUS (DUO92004 – Sigma-Aldrich) PLA probes in 1% BSA for 90 minutes at 37°C. The body walls were then washed twice with Wash A for five minutes each, and incubated in Ligation solution (DUO92008 – Sigma-Aldrich) for one hour at 37°C. Following two washes with Wash A for two minutes each, the body walls were incubated in Amplification solution (DUO92008 – Sigma-Aldrich) for two hours at 37°C. The body walls were then washed twice with Wash B for ten minutes each, followed by a single wash with 0.01× Wash B for one minute. The stained body walls were stored in Duolink In Situ Mounting Medium (DUO82040 – Sigma-Aldrich) at −20°C until ready for imaging.

### Fluorescent *in situ* Hybridisation

FISH was performed as previously described (Lecuyer et al., 2007; Speese et al., 2012). DIG-labeled *par-1* antisense probe was made with the RE47050 cDNA clone by linearizing the plasmid with NotI enzyme and transcribing the cDNA insert with T3 polymerase. Similarly, DIG-labeled *camkII* antisense probe was made with the IP15240 cDNA clone by linearizing the plasmid with EcoRI enzyme and transcribing the cDNA insert with Sp6 polymerase. Both cDNA clones were from the *Drosophila* Genomics Resource Center. Stained body walls were stored in VECTASHIELD Mounting Medium with DAPI.

### GST Constructs

Coding sequences for full-length Hts (1–2154 nts), HtsΔMARCKS (1–2061 nts) and the MARCKS domain (2062–2154 nts) from SD02552 were PCR cloned in frame into pGEX-4T-1 (28-9545-49 – GE Healthcare) using the EcoRI and XhoI sites. Preparation of GST-tagged proteins in BL21(DE3) cells was performed as previously described (Rebay and Fehon, 2009). The GST-tagged proteins were purified with Glutathione Sepharose 4B (17-0756-01 – GE Healthcare), and then eluted with 50 mM Tris (pH 8.0) containing 50 mM reduced Glutathione. Protein concentration was determined using the Bio-Rad Protein Assay.

### PIP Strips

PIP strip membranes (P-6001 – Echelon) were blocked with 0.1% ovalbumin (in TBST - 0.1M Tris, 1.5M NaCl, 0.1% Tween 20, pH 8.0) for two hours. The membranes were then incubated with 0.005 µg/ml of purified GST-tagged protein in 0.1% ovalbumin overnight at 4°C. After three washes with TBST for ten minutes each, the membranes were incubated with primary antibody for two hours. The following primary antibodies were used: 1:1500 mouse anti-Hts-1B1 and 1:10,000 mouse anti-GST (SAB4200237 – Sigma-Aldrich). The membranes were next washed twice with TBST and blocked twice with 0.1% ovalbumin for ten minutes each. The membranes were then incubated with a 1:2000 dilution of peroxidase-conjugated anti-mouse secondary antibody (PI-2000 – Vector Labs) for one hour, followed by four washes with TBST for 15 minutes each. Signal was detected using the BM Chemiluminescence Western Blotting Peroxidase Substrate. Using Photoshop, the film image was inverted and the signal intensity for each lipid spot was measured as mean gray value normalized against the blank. Experiments were done in triplicate with data expressed as absolute values to the control and presented as ‘mean±sem’. Student's t-tests were performed for all statistical comparisons.

## Supplementary Material

Supplementary Material

## Note added in proof

A recent paper has identified adducin as a component of a complex that triggers non-cell autonomous neurodegeneration, particularly in relation to amyotrophic lateral sclerosis (Gallardo et al., 2014).
