# Supplementary material for: Phospho-regulated Drosophila adducin is a determinant of synaptic plasticity in a complex with Dlg and PIP2 at the larval neuromuscular junction
Source: Biol Open. 2014 Nov 21;3(12):1196–206. doi: 10.1242/bio.20148342 (PMC4265757; doi:10.1242/bio.20148342)
Supplement: Supplementary Material [file supp_3_12_1196__index.html]

Phospho-regulated Drosophila adducin is a determinant of synaptic plasticity in a complex with Dlg and PIP2 at the larval neuromuscular junction — Phospho-regulated Drosophila adducin is a determinant of synaptic plasticity in a complex with Dlg and PIP2 at the larval neuromuscular junction — Supplementary Material 

# Phospho-regulated *Drosophila* adducin is a determinant of synaptic plasticity in a complex with Dlg and PIP2 at the larval neuromuscular junction

## bio.20148342 Supplementary Material

**Files in this Data Supplement:**

- Supplementary Material - Simon Ji Hau Wang et al. doi: 10.1242/bio.20148342
