# Supplementary material for: Phospho-regulated Drosophila adducin is a determinant of synaptic plasticity in a complex with Dlg and PIP2 at the larval neuromuscular junction
Source: Biol Open. 2014 Nov 21;3(12):1196–206. doi: 10.1242/bio.20148342 (PMC4265757; doi:10.1242/bio.20148342)
Supplement: Supplementary Material [file supp_bio.20148342_bio.20148342-s1.pdf]

 Biology Open (2014) 000, 1–11 doi:10.1242/bio.20148342

Supplementary Material

Simon Ji Hau Wang et al. doi: 10.1242/bio.20148342

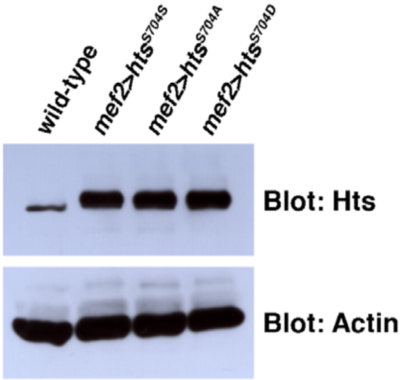

Fig. S1. Expression levels of the wild-type, non-phosphorylatable and phospho-mimetic *hts* transgenes. Western blot analysis of third instar larval body wall lysates immunoblotted with the 1B1 antibody. The *wild-type (UAS-hts^{S704S}), non-phosphorylatable (UAS-hts^{S704A}) and phospho-mimetic (UAS-hts^{S704D}) hts transgenes* were expressed in the muscle with *mef2-Gal4*. All three transgenes show comparable elevations in Hts protein levels over the wild-type control. Actin was used as a loading control.

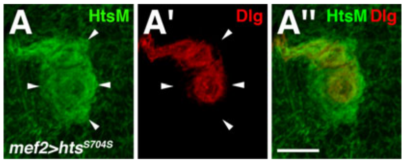

Fig. S2. Co-localization analysis of Hts and Dlg at the postsynaptic membrane of larval NMJs when wild-type Hts is over-expressed in the muscle. (A–A″) High magnification view of a few boutons from a third instar larval NMJ at muscles 6/7 in abdominal segment 4, immunostained with anti-HtsM (green) and anti-Dlg (red). Wild-type Hts is over-expressed in the muscle with *mef2-Gal4*. In many cases, the ectopic accumulation of over-expressed Hts at the NMJ does not exactly match the diffuse distribution of delocalized Dlg (see arrowheads). This indicates that over-expressed Hts does not disrupt Dlg localization by simply 'pulling' it away from the postsynaptic membrane. Scale bar in Panel A″ represents 10 μm.

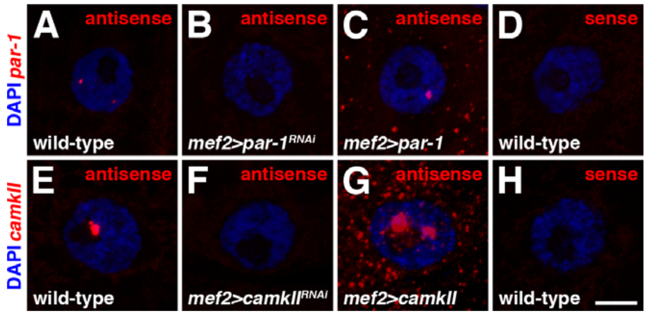

Fig. S3. Specificity of the antisense probes used in the FISH experiments. (A–C,E–G) High magnification views of nuclei (single sections taken within the nucleus) in muscles 6 or 7 in abdominal segment 4, stained with either *par-1* or *camkII* antisense probe (red). Nuclei are marked with DAPI (blue). (A,E) In wild-type, FISH signal is observed in muscle nuclei when using the antisense probes. (B,F) Expression of transgenic *par-1* or *camkII* RNAi with *mef2-Gal4* leads to reduced FISH signal in muscle nuclei. (C,G) In contrast, over-expression of PAR-1 or CaMKII leads to elevated FISH signal in both the muscle nuclei and cytoplasm. (D,H) High magnification views of nuclei (single sections taken within the nucleus) in muscles 6 or 7 in abdominal segment 4, stained with either *par-1* or *camkII* sense probe (red). Nuclei are marked with DAPI (blue). Parallel experiments performed with sense probes resulted in no observable FISH signal in wild-type muscle nuclei. Scale bar in Panel H represents 10 μm (for A–H).

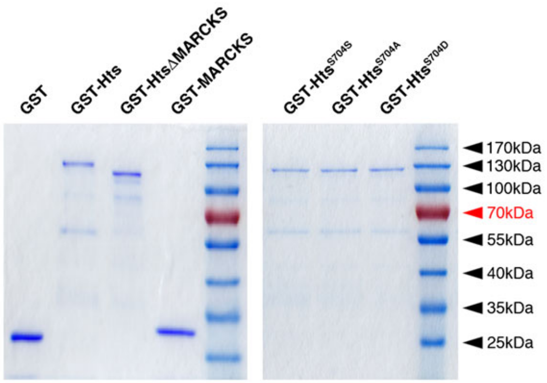

Fig. S4. Expression levels of the GST fusion proteins. Western blot analyses of GST fusion protein levels stained with Coomassie Brilliant Blue. GST fusion proteins were expressed in BL21(DE3) bacterial cells and purified with Glutathione Sepharose. The protein concentration for each eluted GST fusion protein was determined by Bradford assay, and then standardized to 0.005 μg/ml.

Biology Open
